# *Staphylococcus* spp. Epidemiology, Virulence, Genomic Adaptability and Coinfection in Broiler Chickens

**DOI:** 10.3390/ani16020208

**Published:** 2026-01-09

**Authors:** Delvin O. Combar, Sung J. Yu, Emmanuel Asare, Thi T. H. Van, Yadav S. Bajagai, Dragana Stanley

**Affiliations:** 1 Institute for Future Farming Systems, Central Queensland University, Rockhampton, QLD 4702, Australia; delvin.combar@cqumail.com (D.O.C.); s.yu2@cqu.edu.au (S.J.Y.); emmanuel.asare@cqumail.com (E.A.); thithuhao.van@rmit.edu.au (T.T.H.V.); y.sharmabajagai@cqu.edu.au (Y.S.B.); 2 School of Science, RMIT University, Bundoora West Campus, Bundoora, VIC 3083, Australia

**Keywords:** coinfection, bacterial chondronecrosis with osteomyelitis (BCO), biofilm formation, coagulase-negative staphylococci (CoNS), whole-genome sequencing (WGS)

## Abstract

*Staphylococcus* species are saprophytic, opportunistic, and nosocomial pathogens that frequently co-infect with other microorganisms, causing severe infections in birds. Some of the notable examples include bacterial chondronecrosis with osteomyelitis (BCO), cellulitis, dermatitis, and systemic infections. Understanding of how *Staphylococcus* spp. cause infections evading the host immune system is crucial for helping farmers and veterinarians develop long-term solutions for poultry production system management. The aim of this review is to broaden the understanding of *Staphylococcus* spp. epidemiology, virulence, genomic adaptability and coinfection patterns. The peer-reviewed articles were obtained from various databases, including Google Scholar, Web of Science, and PubMed. The review primarily focused on papers published between 1999 and 2025. The review presents an opportunity to identify research gaps and apply this knowledge to develop innovative approaches to address staphylococcal infections in broiler chickens. Additionally, BCO is often attributed to coinfection with *Staphylococcus* species and other pathogens.

## Simple Summary

*Staphylococcus* species are widely distributed in the environment and contribute to a significant number of infections in broiler chickens. This review provides insight into the epidemiology of staphylococcal pathogens and the role of farm management, hygiene, and bird immunity in pathogen control and disease prevention. Advances in sequencing-based molecular diagnostics are challenging the one pathogen—one disease paradigm, with increased reports of coinfections favouring a concept of the pathobiome rather than the pathogen. We focused on virulence, adaptability, and coinfection.

## 1. Introduction

*Staphylococcus* species are widely distributed in the environment, including soil, water, and air, and can also be isolated from animals, such as broiler chickens [1]. Broiler chickens are bred explicitly for extremely rapid growth, high meat yield, and highly efficient feed conversion. Reaching market weight in just weeks, they are exposed to significant environmental stresses, including temperature extremes, high stocking densities, poor ventilation, and frequent disease outbreaks, making them highly susceptible to pathogens. They are saprophytic, opportunistic, and nosocomial pathogens [2,3] that can inhabit the skin and mucosal surfaces of both humans and animals [2,3,4]. Further, enterotoxigenic strains are responsible for staphylococcal food poisoning by producing staphylococcal enterotoxins in food [5,6,7,8]. Therefore, investigating staphylococcal pathogens is essential to mitigate their negative impacts on farms, with a specific focus on broiler chickens.

The genus encompasses approximately 60 known species [9]. They were traditionally divided into two groups based on their ability to coagulate plasma [1], and they are categorised as coagulase-positive staphylococci (CoPS) and coagulase-negative staphylococci (CoNS) groups. Clinical studies have shown that CoPS are highly pathogenic, including *Staphylococcus hyicus*, *Staphylococcus aureus*, *Staphylococcus intermedius*, *Staphylococcus schleiferi*, *Staphylococcus pseudointermedius*, *Staphylococcus lutrae*, and *Staphylococcus delphini*. *S. aureus* draws considerable interest in infection studies since the clinical strains can cause infections in several organs and produce ~25 different toxins associated with severe food poisoning [10]. *Staphylococcus* species are specifically associated with several infections in broiler chickens, including septicaemia, endocarditis, synovitis, and arthritis. Most staphylococcal infections originate from minor skin problems that can spread and, in severe cases, develop into life-threatening conditions.

CoNS are less virulent and are generally considered opportunistic pathogens primarily affecting immunocompromised hosts. The pathogens form part of the normal microbiota of both humans and animals and are responsible for colonising the gastrointestinal and respiratory tracts, skin, and mucous membranes [1]. However, further investigation into their involvement in both human and animal infections has sparked increased research interest in the past decade [11].

CoNS are increasingly linked to significant clinical conditions in broiler chickens, including cellulitis, hepatic and pulmonary granulomas, gangrenous dermatitis, and subcutaneous abscesses [1]. Critically, other CoNS species such as *Staphylococcus xylosus* and *Staphylococcus simulans* are associated with severe bone infections and endocarditis [12,13,14]. The growing body of evidence moves CoNS beyond simple contamination, warranting increased research interest in their involvement in animal infections. The pathogens may also cause subclinical disease, resulting in microscopic lesions in the liver, spleen, and intestines [15,16,17]. Furthermore, CoNS produce fewer toxins and extracellular enzymes than *S. aureus*, and their pathogenicity is mainly associated with cell-surface structures and extracellular components, such as adhesins and mucus layers, which support colonisation of organs [18]. Previous studies have considered CoNS nonpathogenic and the recovery of the group in hosts as contamination.

Several species, including *Staphylococcus gallinarum*, *Staphylococcus arlettae*, *Staphylococcus chromogenes*, *Staphylococcus xylosus*, and *Staphylococcus epidermidis*, have been recovered from the skin and nasal passages of healthy chickens and implicated in cases of dermatitis, tendinitis, and endocarditis [19]. They also cause contamination of chicken meat, mainly due to poor carcass handling or cross-contamination during processing [20]. Recently, CoNS species, including *Staphylococcus equorum*, *Staphylococcus saprophyticus*, *Staphylococcus haemolyticus*, *S. xylosus*, and *S. epidermidis*, have been increasingly isolated from human clinical samples [21,22]. However, due to their major pathogenic potential and genetic variability, *S. epidermidis* and *S. saprophyticus* are clinically highly relevant. *S. epidermidis* strains exhibit significant genetic diversity depending on their source, and isolates from catheter-related infections differ genetically from those found in other environments [23].

## 2. Lameness and Skeletal Diseases in Broiler Chickens

Among the most prevalent challenges in the poultry industry are skeletal disorders affecting the legs and reducing locomotive ability [13,24,25]. Lameness has a negative impact on farmers, causing economic losses by affecting both birds and production efficiency, resulting in higher feed conversion ratios (FCR), reduced growth performance, increased culling, and increased mortality. The susceptibility of fast-growing broilers to lameness is due to the inability of their skeletons and bone strength to keep pace with rapid muscle growth and body weight gain [26,27]. This intense genetic selection for rapid muscle mass accumulation places excessive pressure on the skeletal system, leading to bones that are under-mineralised, porous, and prone to mechanical stress. This trauma, coupled with systemic infection, is key to the subsequent development of lameness and osteomyelitis. Chickens with severe infections have difficulty accessing the drinkers and feeders. Subsequently, compromised welfare affects the quality of chicken products and reduces productivity in birds [28,29,30,31].

Specifically, economic losses from skeletal problems in birds persist for decades. In the early 1990s, the U.S. suffered significant losses in the broiler industry, estimated at $80–120 million, and in turkey production, estimated at $32–40 million [32,33]. Bacterial chondronecrosis with osteomyelitis (BCO), a very common infection among birds, accounted for 0.75% of broiler placements, costing the UK industry £3 million annually at the time [13,32,34]. Another 0.5–0.7% loss was attributed to culling and mortality, equivalent to a loss of 3.75 million birds valued at £4.7 million annually [32,35]. Globally, approximately 12.5 billion broilers develop leg and skeletal problems each year [36], and reports by the European Commission indicate that about 30% of broilers raised in intensive systems suffer from leg disorders [37,38,39,40]. In Australia, limited data are available on the impact of *Staphylococcus* species on broiler chickens, but reports of lameness attributed to BCO have led to culling in broiler flocks. Understanding *Staphylococcus* pathogenicity will help develop strategies to manage disease, reduce human infections associated with chicken meat consumption, increase productivity, and reduce economic losses in the poultry industry.

Staphylococci are commonly found on poultry farms and in hatcheries and can contaminate poultry feed, egg incubators, and hatchers [41,42]. Pondit et al. [43] highlighted a study on chicken table eggs, which detected *Staphylococcus* spp. and *S. aureus* on the eggshell surface in approximately 20.45% and 10.45% of the samples, respectively. The eggshell reflects both the faecal microbiota of the laying breeder chickens and the surrounding environment [44,45], frequently containing Staphylococcaceae. However, recent data could not confirm the presence of eggshell microbiota, feed, and drinking water in the intestinal colonisation of newly hatched chicks [46].

## 3. Virulence Factors

Staphylococci possess a wide array of virulence factors that allow them to colonise hosts, evade the immune system, cause tissue damage, and form biofilms, all of which contribute significantly to their pathogenicity [32]. Genomic studies are vital for identifying these factors, as differences in gene patterns often reflect the host origin and pathogenic potential of the isolates.

### 3.1. Adhesins

Staphylococci’s ability to colonise bones and joints depends on their attachment to the host’s extracellular matrix (ECM). Adhesion initiates the first step in successful infection via adhesin proteins [32]. Staphylococcal adhesins are categorised into two main groups based on their location and mechanism of attachment to microbial surface components recognising adhesive matrix molecules (MSCRAMMs), which are covalently anchored to the bacterial cell wall [47,48], and the secretable expanded repertoire adhesive molecules (SERAMs), which are secreted into the extracellular environment [49,50,51]. MSCRAMMs associated with bone and joint infections are fibronectin-binding proteins (FnBPs), collagen adhesin (Cna), and clumping factors (Clf). Nevertheless, no single adhesin is uniquely associated with bone infections [52]. While adherence-related genes like *atl*, *ebh*, *clfB*, *ebp*, *efb*, *fnbB*, *icaA-D*, *icaR*, and *sdrC* were present in all sequenced *S. aureus* retail meat strains, some adhesins exist in isoforms, including the FnBPs, which occur as FnBPA and FnBPB in *S. aureus* but vary in prevalence within bacterial populations [53].

The proteins bind strongly to fibronectin, fibrinogen, and elastin, and play central roles in adhesion and bacterial internalisation [54]. Further, Cna binds type I collagen and plays a significant role in the development of arthritis, osteomyelitis, and the hematogenous spread of *S. aureus* to bone tissue [55,56]. The *cna* gene was found to be present only in retail turkey isolates in one genomic study [57]. The gene encoding the laminin-binding surface protein enolase (eno) was highly represented in *Staphylococcus* isolates recovered from broiler skeletal lesions, and analysis showed a highly significant association between the *Staphylococcus* species isolated and the *eno* gene [58]. Clf, which also exists in two isoforms (ClfA and ClfB), primarily recognises fibrinogen to facilitate clumping [47]. The virulence factor has been linked with endovascular infections [59,60] and septic arthritis [61].

Other adhesins can manipulate the host’s immune responses, consequently resulting in bacterial internalisation [62,63]. Internalisation is specifically associated with the presence of small-colony variants (SCVs), naturally occurring staphylococcal subpopulations with distinctive phenotypic traits [64]. SCV specifically compromises the host immune system by impeding the activation of key immune responses, lowering endotoxin production, and increasing adhesin expression, thereby facilitating the ability of pathogens to invade host cells over a long period [64]. There is a strong relationship between the emergence of SCV and the ability of bacteria to adapt to hostile environmental conditions, characterised by low pH [65,66], low temperatures, antibiotic treatment [67], nutrient deprivation [68], and oxidative stress [69].

SCVs may revert to their original phenotype when harsh conditions are alleviated, potentially resulting in recurrent and chronic infections. Notably, SCVs exhibit high resistance to antimicrobial treatment [64], rendering them lethal to host cells. Studies have reported SCVs in several notable *Staphylococcus* species, including *S. aureus* and *S. epidermidis* [64,70,71]. However, the prevalence and pathogenic role of SCVs in the broiler industry remain areas requiring further clarification, as current knowledge largely relies on a few experimental cases involving chicken embryo osteoblasts [72]. Given their high resistance to antimicrobial treatment, understanding whether SCVs occur naturally in poultry infections is critical for chronic disease management.

### 3.2. Biofilm Formation

**Figure 1:**
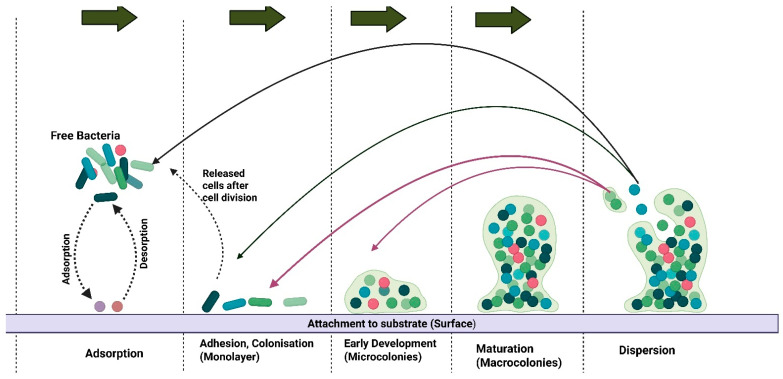
A stepwise illustration of biofilm formation. The colonies attach to the substrate through adsorption and detach through desorption. The macrocolonies are dispersed and attach to other areas, either as free bacteria or as cells released after cell division. Created in BioRender. https://BioRender.com/qnmuega.

Biofilm formation provides an avenue for *Staphylococcus* species to resist antimicrobial activity and evade host defences (Figure 1). In studies focused on skeletal lesions, nearly all (97.8%) *Staphylococcus* isolates formed biofilms, although most were categorised as weak biofilm producers [58]. The ECM, which comprises the biofilm structure, shields the pathogen from phagocytosis and antimicrobial peptides, significantly increasing its tolerance to antibiotics, particularly *β*-lactams and fluoroquinolones [73,74,75]. Biofilm formation by *Staphylococcus* species specifically complicates the treatment of infections in broiler chickens [73] due to difficulties in antimicrobial penetration, thereby increasing the likelihood of chronic and recurrent infections [74]. Therefore, a review of biofilm formation by *Staphylococcus* spp. provides an opportunity for developing solutions to address issues related to pathogen virulence factors.

Pathogens capable of forming biofilms also exhibit higher persistence on abiotic surfaces, including water lines, litter particles, and hatchery equipment [76]. The *icaADBC* operon, encoding polysaccharide intercellular adhesin (PIA) and mediated by intracellular adhesin (ica), plays a central role in biofilm formation [77,78,79]. The gene is regulated by several factors, including SarA, σ^B^, IcaR, and TcaR. Proteins like IcaR repress the *icaADBC* operon transcription to control biofilm formation [79], and σ^B^ regulates *icaADBC* gene directly and indirectly [77]. *Staphylococcus* spp. has IcaZ, which is a non-coding RNA adjacent to the PIA synthesis-mediating *icaADBC* locus [79]. The gene is triggered by environmental stimuli, subsequently activating PIA to initiate biofilm formation. However, *IcaZ* is specifically associated with the *S. epidermidis* ica locus and no other *Staphylococcus* species, including *S. aureus. S. aureus* has both *icaADBC* and *icaR* repressor genes, and this emphasises the differences in structure and organisation of the ica locus in different *Staphylococcus* species [79].

Genetic analysis revealed statistically significant associations between lesion severity and the presence of *icaA* and *icaD* [58,80]. Furthermore, the biofilm phenotype showed a positive correlation with the laminin-binding protein gene *eno* [81]. In poultry, *S. aureus* isolates associated with sternal bursitis demonstrated moderate biofilm-forming capacity, and this ability on damaged bone or joint tissue likely facilitates chronic osteomyelitis and lameness [82]. Regulation is mediated by the accessory gene regulator (*agr*) quorum-sensing system and the staphylococcal accessory regulator (*sarA*) [79,83]. Hence, it is essential to devise strategies to prevent or minimise the formation of biofilms by staphylococcal pathogens to reduce infections in broiler chickens.

### 3.3. Immune Evasion

Some staphylococcal pathogens have a mechanism to evade the immune system via the presence of Protein A (Spa) [84], which binds to the Fc region of immunoglobulins, thereby compromising the ability of antibodies to mark pathogens for phagocytosis. The Spa protein, identified by spa typing, exhibits polymorphisms that are crucial for epidemiological investigations [85]. Further, clinical bacteria, such as *S. aureus*, also harbour capsular polysaccharides (CP5, CP8), which protect clinical strains from host immune responses [86,87]. The pathogen has been shown to secrete molecules such as the staphylococcal complement inhibitor (SCIN) [88,89] and the chemotaxis inhibitory protein (CHIPS) [88,90], which interfere with complement activation and inhibit neutrophil activity. For instance, SCIN targets the central complement enzymes C3bBb and C4b2a of the alternative pathway and classical/lectin pathway, respectively, upon assembly on the microbial surface [88]. As a result, there are reduced Cb3 deposits and opsonophagocytic recognition by neutrophils and macrophages, which increases the survival of pathogens on surfaces and biofilms [88].

Comparably, CHIPS specifically blocks the G-protein coupled receptors (GPCRs) on neutrophils and monocytes, predominantly the C5aR1 (CD88) and the formyl peptide receptor (FPR/FPR1), subsequently preventing the binding and activation of respective ligands like C5a and formylated peptides (fMLP) [89]. The blockage of CD88 and FPR1 results in the inhibition of neutrophil chemotaxis, reduction in neutrophil activation, and impaired recruitment of phagocytes to the infected regions. Like SCIN, CHIPS reduces opsonophagocytic clearance while increasing the window for pathogens to spread disease [89]. In a genomic context, the Immune Evasion Cluster (IEC) genes, including *scn*, are often present in human strains, but the absence of *scn* has been noted in poultry-adapted ST5 strains, suggesting that human-specific virulence factors were lost after host jump to poultry [82,91]. Furthermore, virulence genes associated with immune evasion, such as *aur*, *coa*, *sak*, *sbi*, and *scn*, were identified in poultry isolates belonging to ST291 and ST88 lineages [92]. The virulence factors affect the immune system, thereby allowing *S. aureus* to enter systemic circulation and eventually affect the bones [86].

### 3.4. Cytotoxin and Tissue Damage

Several toxins and exoenzymes are also highly expressed in staphylococci, acting as virulence factors that exacerbate infections by impairing the host’s immunity and damaging tissues [93]. Global regulatory systems, such as *agr*, *sarA*, and *sae* [94], control the expression of virulence factors. Importantly, *Staphylococcus* species lacking *agr* or *sae* loci have a limited exoprotein profile, subsequently resulting in diminished bone remodelling and reduced intraosseous survival [95] of the pathogens in murine models [94,95]. *Staphylococcus* spp. also have cytotoxins that contribute to tissue necrosis and bone damage. For instance, α-toxin (Hla) [96] forms a pore in host cell membranes, causing lysis of erythrocytes, leukocytes, and osteoblasts [97].

Also, leucocidins such as LukMF and LukAB specifically target leukocytes, thereby depleting immune effectors [32]. Panton-Valentine leucocidin (PVL) is rare in poultry, but its presence exacerbates pathogenicity [32]. Further, the genus produces exfoliative toxins (ETA, ETB), which are linked to dermatitis and skin lesions, and enterotoxins (SEs) and toxic shock syndrome toxin (TSST-1), which are superantigens that can drive systemic inflammation and septic shock [97]. Comparative analysis identified a specific strain of *S. aureus* responsible for chicken infections since the 1980s. The chicken-specific clade has evolved since, acquiring additional adhesins, novel virulence determinants, and mobile genetic elements, including staphylococcal pathogenicity islands (SaPIs) [32]. A notable SaPI is hypothesised to play a key role in the pathogenesis of BCO and in enhancing the strain’s virulence [98], making toxins and exoenzymes significant virulence factors in the pathogenesis of various infections in broiler chickens.

## 4. Coinfection

**Figure 2:**
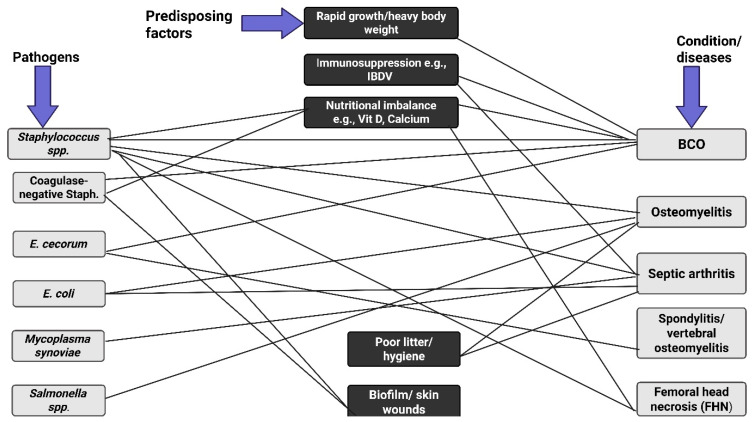
A schematic network of *Staphylococcus* coinfections with some pathogens and the predisposing factors. The disease conditions include bacterial chondronecrosis with osteomyelitis (BOC), osteomyelitis, septic arthritis, spondylitis, and femoral head necrosis (FHN). Rapid growth rate, compromised immune system, poor hygiene, biofilm formation, and nutritional imbalance make the birds vulnerable to pathogenic infection. Proper management of these factors increases the chances of reducing pathogenic invasions. Created in BioRender. https://BioRender.com/fbx6j64.

BCO is currently the most common cause of lameness in broiler chickens globally and often results from coinfection with multiple bacterial genera, including *Staphylococcus* spp., *Escherichia coli*, and *Enterococcus* spp. [99], Figure 2. The increased infections in chickens are due to infections involving primary and secondary pathogens. For instance, BCO was first identified in Australian turkeys in 1972 [38]. The disease was commonly associated with *S. aureus* before traces of *E. coli* were reported [40]. The condition is currently the most common cause of lameness in broiler chickens globally [99] and is described in the veterinary literature as femoral head necrosis (FHN), osteomyelitis, long bone necrosis, degeneration of the proximal femur, and bacterial chondronecrosis (BCN) [12]. Other studies have investigated the disease in chickens, highlighting that the key pathogens are *Staphylococcus* spp., *Enterococcus* spp., *E. coli*, or *Salmonella* spp. [25,38,100,101].

Recent studies have identified *Staphylococcus agnetis*, a coagulase-variable staphylococcal species which was previously linked to dairy cattle mastitis, as an emerging agent in poultry BCO [102]. The pathogen was reported in chickens suffering from BCO in the USA in 2015 [103]. Phylogenetic analysis, based on multi-locus sequence typing (MLST) and genome distance comparisons, strongly suggests that the chicken isolates cluster together, indicating a single, recent host jump of *S. agnetis* from cattle to chickens [34]. In 2017, there was a longitudinal study in Denmark linking *S. agnetis* to 2.7% of deaths attributed to endocarditis and septicaemia. Further study involving the isolation of the microbe from the cloacae of newly hatched chicks suggested that *S. agnetis* could be transmitted vertically from the parent stock to the offspring [104].

Another condition caused by staphylococcal pathogens is spondylitis, a form of BCO characterised by the formation of fibronecrotic fibrosis in the free thoracic vertebra (FTV) [25]. *Enterococcus cecorum* has been reported as a primary cause of spondylitis [105,106,107,108]. However, studies have demonstrated that spondylitis in chickens can be a single or mixed infection involving *S. aureus* and *E. coli* [105]. Studies with *S. epidermidis* (formerly *Staphylococcus albus*) have also confirmed that broiler chickens can develop spondylitis after subsequent inoculation with this pathogen [32].

## 5. Pathogenesis

### 5.1. Bacterial Chondronecrosis with Osteomyelitis (BCO)

BCO is currently the best descriptor of skeletal infections in broiler chickens, as the term reflects observable macroscopic and microscopic changes and identifies the source of the infection [32]. The condition is characterised by a bacterial infection accompanied by necrosis at the proximal ends of the femur and tibiotarsus. The disease can affect one or both limbs and can appear in several skeletal sites within the same bird, with lesions most frequently observed in the femur and FHN [27]. BCO is common in broiler chickens older than 14 days, with the highest occurrence around day 35 [12,25].

There is limited study on the exact mechanism of BCO pathogenesis. However, it is hypothesised that disease development occurs through translocation from damaged skin or mucosal surfaces into deeper tissues and the circulation, omphalitis, vertical transmission through eggs, or infections originating in the respiratory system via the air sacs [12]. The presence of lesions at multiple skeletal sites in a single bird strongly indicates bacteraemia, regardless of the entry route. One leading hypothesis highlights that opportunistic pathogens [1,2,3] enter the bloodstream through the digestive or respiratory tract and colonise the damaged tissues adjacent to the epiphyses [25]. Further, interference with the blood supply at the proximal ends of the long bones, coupled with injury to physeal and epiphyseal cartilages, might play a fundamental role in BCO pathogenesis [25,27,109].

The physeal cartilage receives blood supply from vessels originating from the articular cartilage and metaphysis, which rarely cross the entire physeal cartilage. As a result, the vessels form hairpin loops, leaving part of the physis plate non-vascularised. Blood flow in the vessels is sluggish, and their endothelial linings contain large openings, consequently creating favourable conditions for bacterial colonisation [27]. Loss of the cartilage matrix through microfractures further increases the risk of bacterial invasion. In broiler chickens, rapidly growing plates make chickens vulnerable to *Staphylococcus* spp. infections. For instance, local ischemia and necrosis are consequences of damage to blood vessels near the physis, which creates more avenues for bacterial infection [27]. BCO is commonly associated with compromised immunity, which can be attributed to infectious and non-infectious stressors [39]. Secretion of glucocorticoids is an example of stress-induced secretion, and this has been confirmed through the host immune system suppression in stress-susceptible birds. The susceptibility of broiler chickens to BCO infection may also increase in the presence of predisposing conditions such as tibial dyschondroplasia or rickets, as bone lesions provide an ideal site for bacterial colonisation [28,32,110].

The infection progresses in stages, and pathogenesis begins at the physis. First, femoral head separation takes place (FHS, or epiphyseolysis) [32,111,112], characterised microscopically by irregular articular surfaces and numerous dilated capillaries in vascular channels [109]. Progression of the infection can consequently result in other conditions, such as necrosis, ulceration, and eventual growth plate fracture, a phase often described as femoral head transitional degeneration (FHT) [109]. The final stage of BCO infection is FHN, which is characterised by visible perforations, collapse, and osteomyelitis [38,109]. Similar lesions in the proximal tibiotarsus are referred to as tibial head necrosis (THN) [27].

### 5.2. Spondylitis

Spondylitis is characterised by the formation of fibronecrotic abscesses, and this is among the manifestations of BCO in broiler chickens. The condition mainly develops within the FTV [25,113]. Notably, it is the sole point of connection between the notarium and synsacrum, and the mobility concentrates stress and pressure around the region, resulting in microfractures in the cartilage [25]. Spinal fibrosis is less common in other vertebral regions apart from the FTV, but may develop in young birds since the notarium and synsacrum retain some mobility between their vertebrae, consequently allowing the formation of microfractures in the spinal region [32]. Similarly, spondylitis develops in the spine when opportunistic bacteria circulating in the blood colonise damaged vertebrae, leading to a progressive compression of the spinal cord and demyelination and necrosis of the nervous tissue [114]. The degree of lameness observed in clinical studies always correlates with the extent of spinal cord compression. Diseased chickens rest on the ground with their legs extended forward, a distinctive characteristic of kyphosis (kinky back) in severe conditions [25,27,105].

### 5.3. Synovitis and Arthritis

Synovitis, also known as active inflammatory arthritis, is among the earliest staphylococcal infections detected in poultry [32,115]. The frequently affected areas are the hock, metatarsal, and toe joints, which become hot, swollen, and painful, and similar problems are observed along the tendons [116,117]. Necropsy investigations show that joints often contain fibrinous or caseous exudates [32]. Diseased birds are characterised by progressive lameness, reluctance to move, and a tendency to sit on their hocks or breasts with dirty and fluffed feathers. *Staphylococcus* spp. can colonise the synovium, contributing to the development of necrotic lesions, heterophil infiltration, and fibroblast proliferation [32,118]. Joint and sheath lesions are predominantly accompanied by BCO, and joint infections are mainly secondary to bone infections. It is suggested that the pathogenesis of BCO and synovitis may be similar, and the conditions can be treated as a single disease complex [32].

### 5.4. Diagnosis of Staphylococcus spp.-Associated Diseases in Broiler Chickens

Accurate diagnosis of *Staphylococcus* species infections in broiler chickens is challenging because the clinical signs are often nonspecific, and lesions may overlap with those caused by other bacterial pathogens, such as *E. coli* and *E. cecorum* [32,35]. Nevertheless, precise diagnosis is essential for effective disease management, antimicrobial stewardship, and epidemiological surveillance. The most common clinical indicator of staphylococcal infection in broiler chickens is lameness, particularly in birds between weeks 4 and 7 [32]. Affected birds exhibit reluctance to move, uneven gait, or inability to reach feeders and drinkers. Morbidity is often higher than mortality, but severe cases lead to culling or death due to septicaemia [119]. Other visible signs may include swelling of joints (arthritis), sternal bursitis, and, in cellulitis cases, subcutaneous swellings with discolouration [120]. Further investigation is required because non-staphylococcal pathogens can also cause these signs.

### 5.5. Bacteriological Culture and Phenotypic Identification

Traditional laboratory diagnosis of *Staphylococcus* spp. relies on isolating and culturing samples from bone lesions, joints, liver, or heart. Blood can be plated on blood agar. Confirmatory tests include Gram staining, catalase test, and coagulase test. *Staphylococcus* spp. are generally Gram-positive cocci, facultative anaerobes with sizes ranging from 0.5 to 1.5 µm and appear in irregular grape-like shape in pairs, tetrads, or clusters [121]. The genus is further classified into CoPS and CoNS [122,123,124]. Some *Staphylococcus* species also produce pigmentation and form biofilms, which have clinical significance in the pathogenesis of infections [125]. Notably, phenotypic methods are useful but limited by potential misidentification, particularly in mixed infections, and CoNS speciation often requires sequencing of highly variable genes such as *rpoB* and *sodA*, as phenotypic and 16S rRNA gene sequencing alone are insufficient [126,127].

### 5.6. Molecular and Genomic Approaches

**Figure 3:**
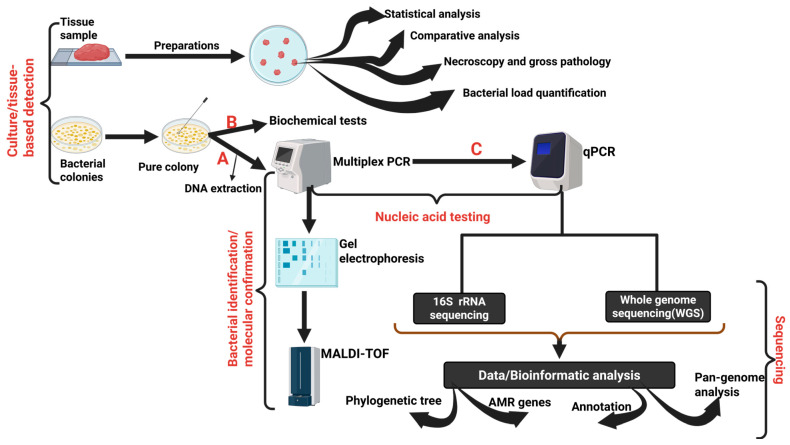
An overview of the steps involved in the diagnosis of *Staphylococcus* spp. infections in broiler chickens. Steps A and B can be performed sequentially, depending on the study’s aim. Steps (C), involving multiplex polymerase chain reaction (mPCR) and quantitative polymerase chain reaction (qPCR). 16S rRNA sequencing and whole genome sequencing (WGS) are conducted to increase the reliability, reproducibility, accuracy, and sensitivity of the diagnostic approach. Created with BioRender. https://BioRender.com/2vhaaxz.

The 16S rRNA sequencing approach has played a fundamental role in investigating the phylogenetic relationships of clinical and non-clinical strains (Figure 3); however, the technique is highly conserved with the 16S rRNA, which limits the accuracy of the approach [128,129]. As a result, other avenues, including the incorporation of MLST to analyse housekeeping genes, have provided pathways to investigate *S. aureus* clonal complexes [130,131]. Other molecular methods, such as spa typing, which relies on polymorphisms in the protein A gene, and pulsed-field gel electrophoresis (PFGE), have proven crucial in epidemiological investigations [132,133]. However, genetic studies involving *Staphylococcus* species have advanced from traditional approaches to novel methods, including whole-genome sequencing (WGS) coupled with bioinformatic studies, resulting in increased accuracy and speed (Figure 3) [134,135,136].

WGS, single-nucleotide polymorphism (SNP) analysis, and genomic island profiling provide unparalleled resolution in outbreak tracking and evolutionary studies [137]. Novel studies incorporating bioinformatic techniques highlight that *Staphylococcus* species genomes range from 2.5 to 3 million base pairs with approximately 30–40% guanine-cytosine [138,139,140,141,142,143], a relatively low GC composition which is characteristic of the genus, consequently acting as a foundational molecular signature. Furthermore, WGS reveals that the genus has compactly arranged genes, enabling rapid adaptation through horizontal gene transfer and the acquisition of genetic elements [144,145]. *Staphylococcus* spp. also possess virulence determinants that help them evade host defences and cause disease [146,147]. Therefore, WGS is increasingly used in epidemiological studies because it can help investigate species identity and provide insights into clonal lineages, virulence factors, resistance genes, and host-adaptation signatures [148].

### 5.7. Histology

Most diagnostic techniques used in bacterial studies are similar, differing only in optimisation and protocol design. For instance, the diagnosis of staphylococcal infections in broiler chickens predominantly relies on flock history and necroscopy studies to assess macroscopic lesions. The lesion patterns may vary, but cases of endocarditis and osteomyelitis at the FTV [106] remain signs of infection in broiler chickens. During the septic stage, a symptom like lameness may be subtle, with minimal clinical signs and limited macroscopic lesions [106]. Collection of samples from key areas, such as joints, pericardium, and bone marrow, enhances the likelihood of detecting *Staphylococcus* spp. These methods strongly support a diagnosis, although the disease may persist subclinically, as the septic phase presents mild or non-evident symptoms.

The septic phase is characterised by both clinical and subclinical signs, with some flocks remaining asymptomatic. In contrast, others exhibit lethargy, fluffed feathers, stunted growth, decreased productivity, and increased mortality, all stemming from widespread infection. Outbreaks are marked with increased mortality from sepsis in early stages, followed by dehydration and starvation in birds paralysed by skeletal complications. The typical cause of the disease is sepsis in the early stages, which progresses to skeletal complications that persist throughout the entire growth cycle [149]. Postmortem examinations reveal that severe pericarditis is characterised by pericardial inflammation and fibrin buildup around the heart, which impairs cardiac function and increases death rates [150]. There is evidence of chronic inflammation of the pericardial and epicardial layers after histological evaluation of the heart and pericardium. The layers are with thickened, hyperplastic, and hypertrophic mesothelial cells [151]. The layers increase in thickness due to the accumulation of oedematous and highly vascular granulation tissue. Scattered fibrin deposits are located on the epicardial and pericardial surfaces and embedded within the fibrous tissue. The infection in the area results in infiltration by intact and degenerate heterophils, macrophages, lymphocytes, and fewer plasma cells throughout the pericardium and epicardium, extending into the outer myocardium. The problem can vary from moderate to severe depending on the level of infection [151]. Necrotic debris is also observable focally within the pericardial cavity.

Liver inflammation (hepatitis) may also be present, with enlarged livers and necrotic lesions contributing to systemic infection [151]. Spleen enlargement and mottling are also indicative of a vigorous immune reaction, which is common with isolates from the spleen, liver, and heart [150,151]. Further, heart tissue may show localised necrosis and pericarditis involving inflammatory cells like neutrophils, histocytes, and lymphocytes [150]. The septic phase allows pathogens to move from the gut into the skeletal structures [152]. Pathogens such as enterococci and staphylococci target the FTV and the femoral head during the skeletal phase [153,154]. Lameness is the first sign, characterised by progressive ataxia resulting from the development of an inflammatory mass at the FTV [153]. Birds become increasingly uncoordinated, progressing to full paralysis, and are mostly seated on their hocks with their legs extended forward [107]. At necropsy, *S. aureus* infections typically present with characteristic BCO lesions in the femoral head and proximal tibia, including necrotic bone and purulent exudates. Microscopically, FHN is characterised by eosinophilic areas containing cellular debris, necrotic chondrocytes, fibrin deposits, and large interalesional cocci colonies, often resembling caseating granulomas [58]. Importantly, CoNS were successfully isolated from macroscopically normal femora and tibiotarsi in infected flocks, suggesting systemic spread even without overt gross lesions.

### 5.8. Differential Diagnosis

There is a notable challenge in distinguishing *Staphylococcus*-related lesions from those of other pathogens, such as *E. cecorum*, since conditions like lameness or septicaemia can produce similar osteomyelitis lesions. Pathogens like *E. coli* are also associated with coinfection with other bacteria, which consequently complicates the investigation of the clinical bacteria causing a particular infection in broiler chickens. Further, infections like viral arthritis caused by reoviruses can mimic *S. aureus*-associated joint lesions [155]. Therefore, combined diagnostic approaches, gross pathology, bacteriology, molecular tools, and epidemiology are essential for accurate investigation of infections in diseased birds. Differential screening using PCR or RT-PCR for co-pathogens like *Mycoplasma synoviae*, *Mycoplasma gallisepticum*, and Avian Reoviruses (ARVs) is crucial, as these co-pathogens can cause septic arthritis or tenosynovitis [156]. Overall, diagnosing *Staphylococcus* infections in broiler chickens requires a multifaceted approach. While field signs and necropsy provide initial suspicion, confirmation relies on bacteriological culture and modern molecular tools such as PCR and WGS. The overlap with other pathogens and the growing importance of antimicrobial resistance surveillance necessitate the use of integrated diagnostics to guide control strategies and protect both poultry and public health.

### 5.9. Potential Routes of Transmission

The transmission of *Staphylococcus* species in broiler chickens is a complex process, involving multiple ecological niches, host reservoirs, and environmental pathways. Understanding these transmission routes is essential for implementing targeted biosecurity and disease control measures. The primary route of transmission in poultry is horizontal spread, which occurs through bird-to-bird contact or via the shared environment. *S. aureus* colonises the skin, respiratory tract, and gastrointestinal tract of apparently healthy birds, serving as a constant reservoir [99]. Contamination can occur during egg handling or incubation, as well as on hatchery surfaces such as trays, chick boxes, and conveyor belts, which can harbour persistent biofilms [157]. Chicks exposed to pathogens at hatching can carry the bacteria throughout life, increasing the risk of systemic disease under stress [157]. Studies on vertical transmission from hen to egg yolk and embryo have not conclusively demonstrated the contribution of *Staphylococcus* species in broiler chicken infections. Hypothetically, trans-shell contamination during laying and incubation is a plausible mechanism for early infection. Therefore, hatchery sanitation and egg-handling practices are critical to controlling or preventing staphylococcal infections.

Minor skin injuries such as scratches, footpad dermatitis, and hock burn also provide portals of entry for *S. aureus*. The bacterium spreads systematically through the bloodstream. Similarly, microfractures in rapidly growing broiler bones predispose the birds to conditions like osteomyelitis [52,158]. The lesion-based routes highlight the interconnectedness of husbandry practices, which encompass litter quality, stocking density, and growth rate, to the infection dynamics in chickens. Furthermore, the transmission route involves interaction between humans and poultry (a zoonotic cycle) that leads to *S. aureus*-related infections. Studies have shown that human-adapted lineages, particularly clonal complex 5 (CC5), can successfully cross into poultry populations and subsequently adapt to the avian host, representing a human-to-poultry transmission [157]. Conversely, poultry workers, veterinarians, and abattoir staff are at risk of acquiring livestock-associated methicillin-resistant *Staphylococcus aureus* (LA-MRSA), especially ST398 and ST9, through direct contact or inhalation of dust [159]. Retail meat isolates from chickens often share MLST type ST5 and spa type t002, aligning them with poultry-associated strains, whereas turkey isolates are more likely to belong to ST398, a dominant LA-MRSA type [160]. The presence of an enterotoxigenic strain of *S. aureus* in contaminated poultry products poses a significant public health risk.

In general, the transmission of *Staphylococcus* species in broiler chickens is multifactorial, encompassing horizontal spread within flocks, hatchery contamination, entry points for lesions, zoonotic cycles, and inter-farm dissemination. Horizontal routes are common in poultry systems, but cases of early chick colonisation and human–poultry exchange can complicate control efforts. Breaking these transmission cycles requires integrated biosecurity, improved hatchery sanitation, and surveillance that recognises the interconnectedness of poultry health and public health.

### 5.10. Antimicrobial Resistance (AMR)

*Staphylococcus* species are generally considered harmless members of the normal microbiota in animals and humans; however, their capacity to acquire antimicrobial resistance (AMR) is a major global health concern [161]. CoNS specifically lack most virulence factors associated with *S. aureus* apart from the toxins and exoenzymes [162]. Nevertheless, the CoNS group is lethal due to its possession of several antimicrobial resistance genes located on mobile genetic elements (MGEs) [163,164,165]. Previously, the exchange of microorganisms and antimicrobial resistance between hosts and environments was overlooked; however, strains recently isolated from both humans and animals highlight the shared resistance genes [166]. Notably, *Staphylococcus* species that are not particularly pathogenic can pose a significant challenge to broiler chickens by potentially transferring resistance genes to more virulent pathogens, especially *S. aureus* [161], thereby increasing resistance to treatment [167] for certain infections. Therefore, some CoNS are potential reservoirs for antimicrobial resistance genes within the *Staphylococcus* genus [118,168].

The acquisition of AMR genes in *Staphylococcus* species is primarily mediated by conjugation, bacteriophage transduction, and the presence of multiple insertion sequences within staphylococcal genomes [161]. The elements are instrumental in genomic rearrangements, which, in turn, result in genetic plasticity and phenotypic diversity across *Staphylococcus* species [167,169]. Historically, bacteriophage transduction was considered the primary route of horizontal gene transfer in staphylococci. Comparatively, conjugation was perceived to play a role in evolution due to the scarcity of mobilisation and conjugation loci in staphylococcal plasmids [167,170]. Approximately 5% of sequenced staphylococcal plasmids contain the genes necessary for autonomous transfer by conjugation. The specifics contrast with the studies on the horizontal transfer of plasmids among different staphylococcal lineages and species [170]. However, an alternative mechanism involved in the transfer of staphylococcal plasmids has been identified within the genus. The approach involves conjugation, which is mediated by integrative and conjugative elements (ICEs), also known as conjugative transposons [171].

Another contributing factor to the increase in resistant *Staphylococcus* strains is the Staphylococcal Cassette Chromosome *mec* (SCC*mec*), which is a mobile genomic island that confers resistance to methicillin and most β-lactam antibiotics [161]. The evolution of SCC*mec* involves recombination and assembly with other staphylococcal species, involving SCC*mec* type III cassette of *Mammaliicoccus sciuri* (formerly *Staphylococcus sciuri*) strains, *Staphylococcus vitulinus*, and *Staphylococcus fleurettii* [161]. The elements were later transferred to *S. aureus* and other *Staphylococcus* species, with CoNS acting as primary reservoirs [172,173].

A group of CoNS has also demonstrated the ability to transfer high-molecular-weight plasmids carrying the *mupA* gene, which confers mupirocin resistance. The transfer from *S. epidermidis*, *S. aureus*, and *S. haemolyticus* strains to another *S. aureus* strain [174,175]. Similarly, Cafini et al. [176] showed that linezolid resistance was mediated by *cfr*-carrying plasmids between *S. epiderdimis*, *S. aureus*, and *Enterococcus* spp. isolate. Furthermore, *Enterococcus* species act as nosocomial pathogens and have been implicated in plasmid exchange with RSA, facilitating the acquisition of vancomycin resistance, which is a last-resort antibiotic treatment option [177]. Meric et al. [178] revealed that *S. aureus* and *S. epidermidis* share approximately half of their pan-genome, but exhibit significant overlap in mobile genetic elements, especially those associated with pathogenicity islands and the SCC*mec* [161]. Biofilm formation by CoNS provides a conducive environment for horizontal gene transfer [179,180,181], thereby increasing the likelihood of infections and antimicrobial resistance.

Biofilm formation, a critical player in bacterial virulence [161], provides an avenue for *Staphylococcus* species to resist antimicrobial activity and evade host defences [182]. There is a limited understanding of how biofilms confer resistance to host defences, despite the identification of several processes. For instance, it is hypothesised that biofilm can restrict leukocyte access to bacterial cells, consequently suppressing the immune system and promoting cell–cell communication to enhance resistance [183]. Reduced membrane permeability also decreases antimicrobial penetration [184]. Notably, antibiotics targeting the *Staphylococcus* cell wall modulate natural transformation through a sigma factor H, SigH-dependent pathway. The activation of SigH-regulated genes facilitates competent transformation of *S. aureus* cells via plasmids or chromosomal DNA, thereby promoting plasmid exchange within biofilms. Additionally, others [185] noted that several antibiotics induce *ccrC1* gene expression, which mediates the excision of the SCC*mec* element from the chromosome, consequently facilitating its transfer.

*S. aureus* specifically undergoes cell lysis during biofilm maturation, which releases genomic DNA that becomes extracellular DNA (eDNA). eDNA is a fundamental component of the biofilm matrix [186], which adheres to cell surfaces in long, loop-like structures, facilitating cell attachment and influencing bacterial cell surface hydrophobicity [187]. Additionally, eDNA reduces vancomycin penetration in *S. epidermidis* by binding to the positively charged antibiotic molecules [188]. eDNA also facilitates horizontal gene transfer by transforming competent cells [189,190]. Although CoNS lacks most virulence factors present in *S. aureus*, biofilm formation remains a defining trait of *S. epidermidis*, *S. haemolyticus*, *S. saprophyticus*, *Staphylococcus hominis*, and *Staphylococcus cohnii.* The feature is closely associated with the high prevalence of antimicrobial resistance among these organisms [163].

### 5.11. Novel Prevention, Control, and Management Strategies

Currently, several challenges associated with antimicrobial use necessitate adopting alternative, innovative methods to manage and prevent the spread of *Staphylococcus* spp. infections in farms globally. Notably, the risk of *Staphylococcus* infections can be reduced by adopting strict biosecurity measures, maintaining proper hygiene, and implementing effective flock management, which subsequently helps address stress and injuries [191] in broiler chickens. For instance, farmers should incorporate measures to limit visitor access to the farm, use clean clothing and footwear, and observe hand hygiene, thereby preventing the spread of pathogens by humans [191]. Specifically, stress and injury management in broiler chickens can help reduce the risk of *Staphylococcus* by preventing skin breaks, gentle handling of the birds, and optimising nutrition and the environment. These approaches can help reduce the incidence of systemic infections, such as BCO [35]. Further, studies should focus on targeted surveillance and diagnostics, antimicrobial stewardship, and other alternatives, such as probiotics and vaccines [191].

Alternative approaches, such as probiotics, are currently being considered to manage staphylococcal infections among farmers. The approach increases the chances of improving gut health, consequently reducing pathogen loads in poultry production [192,193]. However, the effectiveness of probiotics depends on dosage, timing, and the strains used [193]. Bacteriophage therapy is another experimental approach that can help reduce bacterial load. Bacteriophage therapy can be a viable alternative for treating multidrug-resistant pathogens, including *S. aureus*. The *Staphylococcus* species management alternative still has issues related to phage specificity and formulation [194,195]. Currently, the phage spectrum is still narrow, limited to a single bacterial species, which limits the full implementation of the approach. Another notable challenge of phage therapy is the potential for bacterial gene transfer during phage administration and the difficulty of monitoring bacteriophages that can switch between lytic and lysogenic life cycles, thereby increasing the risk of contaminating phage drug products [195]. Nevertheless, bacteriophage therapy offers a promising approach to treating staphylococcal infections in the poultry industry if the gaps in the technique are addressed.

The use of antimicrobial peptides (AMPs) is another innovative therapeutic option with the growing cases of antimicrobial resistance by *Staphylococcus* spp. pathogens [196]. AMPs represent the host’s first line of defence and can act against a wide range of pathogens, including Gram-negative and Gram-positive bacteria, fungi, and viruses. The technique is actively being investigated in humans, and the same approach can be replicated to provide solutions in the poultry industry. AMP activity depends on the lipid type in the bacterial cell membrane [197]. The approach specifically targets the bacterial cell membrane, consequently disrupting the inner and outer membranes, and this causes cell death [198] through AMP interaction with the negatively charged cell membrane, DNA, and RNA synthesis, while also facilitating protein inhibition [199,200,201].

AMPs can prevent biofilm formation at early stages by disrupting signalling pathways, thereby promoting the production of guanosine tetraphosphate (ppGpp) and guanosine pentaphosphate (pppGpp), which limit nutrient availability and inhibit nucleic acid synthesis [196]. Therefore, AMPs present an avenue to counter some of the drawbacks associated with conventional antimicrobials. However, the approach still has issues encompassing low bioavailability and low metabolic stability, especially in oral formulations [202], while the intravenous formulation undergoes proteolytic cleavage in the blood and liver, resulting in reduced effectiveness of AMPs [199]. Therefore, addressing some of these gaps can help develop practical, long-term approaches to tackle pathogens affecting both the poultry industry and public health.

## 6. Implication in the Poultry Industry and Conclusions

*Staphylococcus* species have emerged as a significant pathogen in broiler chickens, with increasing reports of septicaemia. The underlying mechanisms of spread and transmission remain poorly understood, beyond the identified virulence factors in pathogenic strains. Therefore, investigating the transmission dynamics, including both horizontal and vertical transmission, is crucial for developing effective control measures. Lameness and skeletal disorders, especially BCO, rank among the most significant challenges in broiler chickens, compromising bird welfare and contributing to economic losses in the global poultry industry. Intensive genetic selection produces fast-growing broilers with rapidly accumulating muscle mass, which places excessive pressure on the skeletal system at an early age. Stress results in epiphyseal trauma, creating favourable conditions for hematogenous bacterial spread and colonisation. There are several challenges associated with managing staphylococcal infections, given their ubiquity in poultry farm environments. The genus includes explicitly opportunistic pathogens that exhibit broad antimicrobial resistance.

Also, there is an issue with SCV, which is challenging to address when dealing with *Staphylococcus* species. The prevention strategies must target both the variant strains and the host, in addition to the known pathogenic strains. Further, the extensive management of *Staphylococcus* infections in broiler chickens must address direct and indirect transmission pathways, with a specific focus on late-embryonic-phase infections and on vigilant monitoring of emerging resistant clones and zoonotic threats. Conventional antibiotic treatments offer limited benefits, necessitating cautious use to prevent the further development of antibiotic resistance. Management approaches that focus on slowing growth rates have proved beneficial for reducing lameness cases while minimising stress and exposure to immunosuppressive factors, which are critical for reducing susceptibility to staphylococcosis.

## Data Availability

No new data were created or analyzed in this study. Data sharing is not applicable to this article.

## Abbreviations

AMP	Antimicrobial Peptides
AMR	Antimicrobial Resistance
BCO	Bacterial Chondronecrosis with Osteomyelitis
Cna	Collagen adhesin
Clf	Clumping factors
CoNS	Coagulase-Negative Staphylococci
CoPS	Coagulase-Positive Staphylococci
eDNA	Extracellular DNA
Eap	Extracellular Adherence Protein
ECM	Extracellular Matrix
Efb	Extracellular Fibrinogen-Binding Protein
Emp	Extracellular Matrix-Binding Protein
FCR	Feed Conversion Ratio
FHT	Femoral Head Transitional Degeneration
FHN	Femoral Head Necrosis
FnBP	Fibronectin-Binding Protein
IEC	Immune Evasion Cluster
GPI	Glycosylphosphatidylinositol
MSCRAMMs	Microbial Surface Components Recognising Adhesive Matrix Molecules
WGS	Whole-Genome Sequencing
SCIN	Staphylococcal Complement Inhibitor
CHIPS	Chemotaxis Inhibitory Protein
SERAMS	Secretable Expanded Repertoire Adhesive Molecules
SCVs	Small-Colony Variants
THN	Tibial Head Necrosis
